# Beetroot-Carrot Juice Intake either Alone or in Combination with Antileukemic Drug ‘Chlorambucil’ As A Potential Treatment for Chronic Lymphocytic Leukemia

**DOI:** 10.3889/oamjms.2015.056

**Published:** 2015-06-02

**Authors:** Marie-Christine R. Shakib, Shreef G. N. Gabrial, Gamal N. Gabrial

**Affiliations:** *National Research Centre, Nutrition and Food Science Department, El Buhouth St., Dokki, Cairo 12311, Egypt*

**Keywords:** Chronic lymphocytic leukemia, beetroot-carrot juice, chlorambucil drug, leukocytes and lymphocytes count

## Abstract

Chronic lymphocytic leukemia (CLL) is one of the chronic lymphoproliferative disorders (lymphoid neoplasms). It is characterized by a progressive accumulation of functionally incompetent lymphocytes. Patients with leukemia often seek unconventional treatments not prescribed by hematologist in order to improve their cancer treatment outcome or to manage symptoms. In the present report, a 76-year-old patient was diagnosed with B-cell chronic lymphocytic leukemia (B-CLL). Beetroot-carrot juice is used as a complementary and or/alternative therapy used in conjunction with conventional leukemic treatment (chlorambucil) that has been a standard first-line chemotherapeutic agent for patients with CLL and known to have serious and undesirable side-effects. After one month and 15 days of administration of beetroot-carrot juice therapy, the patient had improved appetite, a sense of general well-being and increased vigor daily activities. Furthermore, beetroot-carrot juice was used as an adjuvant to chlorambucil resulted in a substantial reduction in leukocytes and lymphocytes count in peripheral blood and improvement in the relevant biochemical parameters. Beetroot-carrot juice can be used as an effective treatment for CLL alone or in combination with chlorambucil when taken orally with regular diet on daily basis.

## Introduction

Chronic lymphocytic leukemia (CLL) is an indolent lymphoproliferative disorder. It was largely considered to be a disease of slow progression and chlorambucil drug has been the standard treatment for many decades [1]. Most of the anti-cancer drugs currently used in chemotherapy can have serious and undesirable side-effects and may cause toxicity to normal cells. Efforts were made to develop effective alternative strategies that increase the therapeutic efficacy and minimize the systemic toxicity of the conventional chemotherapeutic agents [2]. Thus, natural treatments could support existing chemotherapy agents’ treatment of leukemia, if their use could reduce side effects without compromising efficacy. Promising natural sources of such new agents could be found in common foods which contain bioactive compounds with potential anticancer properties [3]. The beetroot (Beta vulgaris L.), locally known as Shamandar, is a vegetable plant and belongs to family Amaranthaceae. Studies that focused on anticancer activities of beetroot extract, in animal models, have unraveled their potential benefits as chemopreventive and chemotherapeutic agents. Studies by Dr. Sandor (Alexander) Ferenczi (Nobel Prize winner) was pioneered of the use of beetroot juice as a cancer therapy in the nineteenth century and studied its strong anti-tumor effect as it contains a tumor inhibiting substance that he attributes to its natural red coloring agent, betaine [4]. Beetroot has many remarkable therapeutic uses as anticancer, hemostatic, antioxidative, antiradical, anti-inflammatory and renal protective properties. In traditional medicine, carrots (Daucus carota) have been used as treatments for leukaemia and other cancers throughout history [5] and have previously been studied in other contexts as potential sources of anticancer agents [6].

In the current study we present a case report of CLL treated with a blend of beetroot- carrot juice either alone or in combination with antileukemic drug ‘Chlorambucil’.

## Case Presentation

A- 76 -year-old female presented in the year 2010 with a history of fatigue in the last 1-2 years that was progressive and worsened on exertion. At first, the fatigue did not prevent her from doing her daily activities, but later, she became somewhat less active. She was complaining of symptoms of weakness, intermittent upper back pain. She denied having other clinical symptoms as weight loss, night sweats, fevers, cough, chest pain, abdominal pain, dizziness, and syncope. On physical examination, no cervical, axillary, or inguinal lymphadenopathy was observed. No palpable hepatomegaly or splenomegaly was detected. Initial laboratory data undertaken on 8 th January 2010 revealed a high level of Leukocytes and lymphocytes. Haematology report was as follows: Hemoglobin level,11.6 g/dL; Red Cell Count, 3.94 mil/cmm; mean corpuscular volume, 88.3 fL; Leukocytes count, 46,700/cmm.; Lymphocytes, 85%; monocytes, 2.9%, eosinophils, 0.7%; and platelet count, 186 ×103/μL. The patient’s creatinine was normal at this stage (1.04 mg/dL), blood urea was 55 mg/dL.

The patient was diagnosed with B-cell chronic lymphocytic leukemia (B-CLL) based on incidental finding of lymphocytosis, and the typical lymphocyte surface markers with positive CD19, CD5, CD23, and weak expression of surface lambda. Flow cytometry immunophenotyping of peripheral blood, performed in 14 th January 2010 at Quest Diagnostics- West Hills, CA, USA, revealed the presence of an abnormal CD19, CD5, CD23, lymphoid cell population. She was taken to a specialized haematology clinic on 7th April 2010 and the hematologist recommended repeating the immunophenotyping test. The test was repeated in 19 th April 2010 and the phenotypic pattern gives the case a score of ‘5’ in the WHO scoring system for CLL, thus confirming a case of B-Kappa CLL (Table 1).

**Table 1:** Flow cytometry immunophenotyping findings

Study	Results
Flow Cytometry Analysis in 14^th^ January 2010	An abnormal cell population comprises 67% of total cells. The coexpression of CD5 and CD23 in B- cells (CD19+) is consistent with chronic lymphoproliferative disorder (usually chronic lymphocytic leukemia CLL). 0% of the CD19 positive lymphocytes express CD38.
Flow Cytometry Analysis in 19^th^ April 2010	CD19: 90.9%, CD5: 52.7%, FMC7: 11.0%, CD3: 7.65%, CD10:1.15%, CD38: 6.72%, CD23:70.9%, Anti Kappa: 87.9%, Anti lambda:7.57%.
	

As the patient was physically unfit for FCR (fludarabine, cyclophosphamide, rituximab) therapy, the haematologist treated her with single-agent chemotherapy (chlorambucil) as a mild chemotherapy that is currently established as standard treatment [7].

The three treatment phases either with chlorambucil drug, beetroot-carrot juice, or both exhibited different potency:

### Phase 1 - Chlorambucil protocol

**Figure 1:**
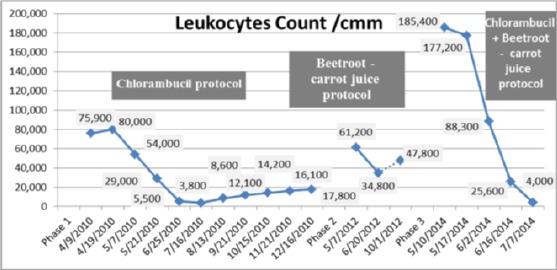
*Change in peripheral leukocytes count during the three treatment phases*.

**Figure 2:**
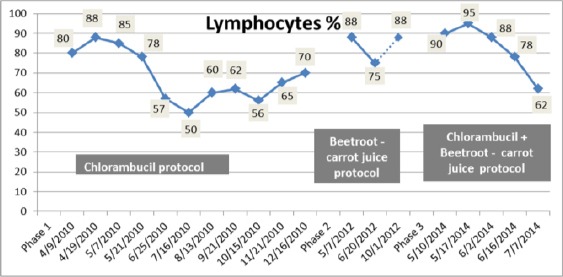
*Change in peripheral lymphocytes count during the three treatment phases*.

The patient received chlorambucil 4 mg/day for a period of 8 months from 19th of April 2010 to 16th December 2010. Total leukocytes count declined from 80,000 to 17,800/cmm (Fig. 1). During the course of the treatment, all CBCs showed absolute lymphocytosis and were not resolved as lymphocytes decreased only from 88 to 70% as demonstrated in Fig. 2. Serum creatinine had fallen from 1.72 to 1.24 mg/dL, but in the middle of the treatment it recorded a high value of 1.86 mg/dL. The patient remained clinically stable but with persistent fatigue.

### Phase 2 (first relapse) - Beetroot- carrot juice protocol

Recurrence of the disease was observed on the 7 th May 2012 with rising count of leukocytes to 61,200/cmm and lymphocytes to 88%. The patient refused to repeat the regimen of the chemotherapy drug chlorambucil and she was convinced to start a new treatment of consuming a natural juice composed of beetroot (Beta Vulgaris L.) blended with carrot (Daucus Carota). The given dose was 330 ml of fresh raw beetroot- carrot juice blend and was administered orally (beetroot 200 g. + carrots 250 g.) two hours before breakfast. The juice taste was palatable and well tolerated. The juice was administered to the patient in daily doses once per day (6 times/week) for duration of 1 month and 15 days. The patient was observed to have an increase in appetite, improvement in performance of daily activities along with lack of fatigue few days after consuming the juice.

In Fig. 1 & 2, it can be seen that by introducing 330 ml of the beetroot- carrot juice to the patient 6 times/week for duration of 1 month 15 days (from 7th May 2012 to 20th June 2012) had the potency to decrease leukocytes count from 61,200/cmm to 34,800/cmm and lymphocytes from 88% to75% on the 20th June 2012. There was also a reduced level of uric acid in blood by 11.57% (9.5 to 8.4 mg/dL).

The patient discontinued the beetroot- carrot juice for a period of 1 month during which she was not cooperative; however on the 20th of July 2012 she resumed the juice. Same dose of the juice was given once per day but only twice/week for a period of 2 months and 11 days till 1st of October 2012.

On the 1st October 2012, leukocytes count started to increase and reached a level of 47,800/cmm and lymphocytes recorded a value of 88%.

### Phase 3 (second relapse) - Chlorambucil + beetroot- carrot juice protocol

The patient stopped the juice regimen for 1 year, 7 months and 9 days as she complained and get bored of drinking the juice daily. The patient presented with a very serious clinical situation. She had fever and the leukocytes count reached a high level. On the 15th April 2014, the patient developed prominent leukocytosis (149,800/cmm) with absolute lymphocytosis (96%). The RBCs and Hb dropped to 2.9 mil/cmm and 9.2 g/dL respectively. Platelet count was 144,000/cmm (normal value). The uric acid reached a level of 9.1 mg/dL. The haematologist has treated the patient with chlorambucil protocol 4 mg/day for a period of one month. The haematologist recommended that the patient should be switched to a salvage therapy if she will not respond to this drug regimen as a single agent as initial therapy.

**Figure 3:**
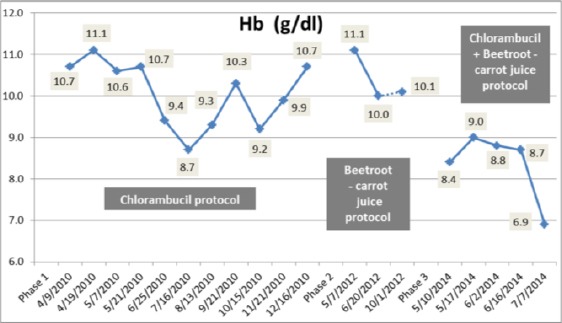
*Change in hemoglobin during the three treatment phases*.

On 10th May 2014, a rapidly rising in leukocytes count occurred with a suppression of haemoglobin (Fig. 3) and platelet production (Fig. 5). The leukocytes count reached a high level of 185, 400/cmm; lymphocytes declined to 90% (Fig. 1, 2) despite the treatment with chlorambucil. The Lactate dehydrogenase (LDH) which is a general indicator of the existence and severity of acute or chronic tissue damage, reached a high level of 360 U/L. The haematologist doubled the dose of chlorambucil to 8 mg/day. The beetroot- carrot juice (330 ml) was then introduced daily; 6 times per week, in combination with the chlorambucil protocol 8 mg/day.

**Figure 4:**
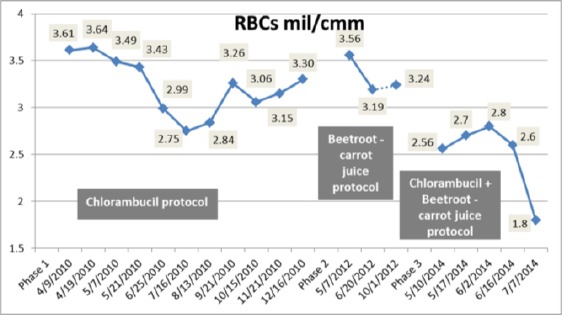
*Change in RBCs during the three treatment phases*.

**Figure 5:**
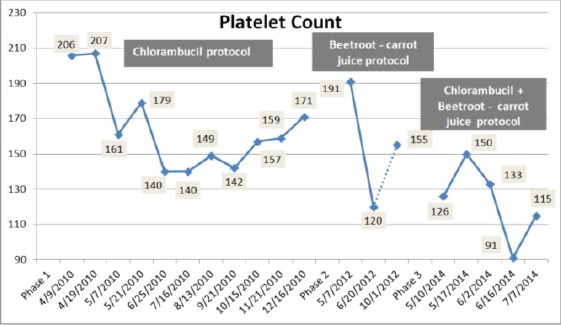
*Change in platelet count during the three treatment phases*.

From the 10th of May to the 16th of June 2014, the patient’s leukocytes count had gradually declined from 185,400/cmm to 25,600/cmm (Fig. 1); lymphocytes count was also reduced from 90% to 78% (Fig. 2). The Hb and RBCs reached a level of 8.7 g/dL and 2.6 mil/cmm, respectively (Fig. 3, 4). Her renal function improved as blood urea, serum creatinine and uric acid dropped from 60 to 52 mg/dL, 1.3 to 1.2 mg/dL and 4.4 to 3.3 mg/dL, respectively. There was no evidence of hemolysis as reticulocytes count decreased from 3.2% to 2.4% and direct Coombs’ test was negative. It was also noticed that LDH decreased to a level of 197 U/L.

During the three treatment phases, hematological parameters including Hb and RBCs recorded low values below normal levels (Fig. 3 & 4), whereas platelets count was fluctuating between normal and below normal levels indicating mild thrombocytopenia (Fig. 5).

The patient refused to cooperate and stopped taking the juice again from August 2014 to December 2014. She followed the initial treatment with chlorambucil alone (8 mg/day) without the juice. A complete regression of CLL documented by a normalization of the peripheral leukocytes and lymphocytes count was observed, but accompanied by deterioration in renal function demonstrated by an increase in serum urea (155 mg/dL) and serum creatinine (1.8 mg/dL). It was unlikely to have been due to uric acid nephropathy as the serum uric acid was not elevated (4.0 mg/dL).

The patient resumed taking the beetroot-carrot juice for one month daily. Kidney function was improved; her serum urea and creatinine level were reduced (77 mg/dL and 1.3 mg/dL, respectively). The total and ionized serum calcium concentration remained normal (8.6 mg/dL and 4.9 mg/dL respectively). Serum Na+ and K+ level recorded normal values (130 mmol/L and 5.1 mmol/L, respectively).

## Discussion

In the present study, the effect of beetroot-carrot juice on leukocytes and lymphocytes count either administered alone or in combination with the antileukemic drug chlorambucil was reported.

From Fig. 1 & 2, we have been able to demonstrate in our patient that administration of beetroot-carrot juice alone (phase 2) induced a reduction in peripheral blood leukocytes and lymphocytes count by 43.13% and 14.7% respectively and a partial remission was achieved 1 month 15 days after the treatment. There was a reduction in the level of uric acid. It was established that the elevated level of uric acid (hyperucimea) was caused by tumor lysis syndrome (caused by the breakdown products of dying cancer cells) of leukocytes cells [8]. Stopping the beetroot-carrot juice for 1 month, then resumption of the juice for another 2 months and 11 days, leads to an increase in leukocytes and lymphocytes counts and increase in uric acid level.

After the second relapse, the patient had not responded to chlorambucil treatment alone from 15th April 2014 till 10th May 2014. In contrast, the combined treatment of the antileukemic drug chlorambucil and beetroot-carrot juice for 1 month and 6 days recorded a significant reduction in total leukocytes and lymphocytes count by 86.19% and 13.3%, respectively. This result provides evidence for the strong effectiveness of the combination treatment of chlorambucil and beetroot-carrot juice in eliminating malignant leukemic cells. Chlorambucil may produce its antitumor effect in CLL by inducing apoptosis-associated membrane changes that resulted in rapid clearance of the apoptotic cells by the immune system [9]. It was shown from our study that beetroot-carrot juice may act in synergism with chlorambucil by lowering peripheral leukocytes and lymphocytes count. Furthermore, the toxic side effects associated with chlorambucil was minimized by combining the dosing regimen (8 mg/day) with the beetroot-carrot juice, building up the patient’s tolerance to the drug. Beetroot -carrot juice reduced the most frequent signs of gastrointestinal tract damage including vomiting, diarrhea, anorexia and stomatitis in response to chlorambucil [10]. Improvement in renal function followed this combined treatment was observed. Therefore, Beetroot-carrot juice presented a successful treatment as it prevented renal impairment due to leukemic infiltration of the kidneys that may result from the use of chlorambucil alone [11].

Many studies showed that red beetroot (Beta vulgaris L.) extract, approved by Food and Drug Administration and European Union as red food color E162, reduced multi-organ tumor formations in various animal models when administered ad libitum in drinking water [12]. Many research exhibited the cytotoxic effects and antiproliferative activity of betanin, the major constituent of the red beet-derived betalains on different tumor cell lines of human origin [13]. It should be noted that betacyanins, among the beetroot betalain constituents, are the most important and potent compound responsible for the observed anticancer effects [14].

Another study reported that betanin, isolated from the fruits of Cactus Opuntia ficus-indica, similar to the betanin isolated from red beetroot [15] induced apoptosis in the human chronic myeloid leukemia cell K562 line [16]. This study have demonstrated that betanin entered K562 cells and made biochemical alterations, and induced the initiation of apoptosis. Furthermore, the high content of folic acid (15.8 mg/g dry matter) is another nutritional feature of beetroot which may account for its anti-proliferative and immunomodulatory activity [17].

Researchers reported the synergistic cytotoxic effect of red beetroot with doxorubicin in human prostate (PC-3), pancreas (PaCa), and breast (MCF-7) cancer cell lines. These results suggested that beetroot extract enhance the therapeutic efficacy of the potent chemotherapeutic drug (doxorubicin) [18].

β-carotene, lutein and polyacetylenes derived from carrot are bioactive compounds shown to have anticancer activities. They have effects on induction of apoptosis and inhibition of cellular proliferation. Treatment of leukemia cell lines with carrot juice extract induced apoptosis and inhibited progression through the cell cycle. Lymphoid cell lines were affected to a greater extent than were myeloid cell lines and normal hematopoietic stem cells were less sensitive than most cell lines [19].

In the present study, red blood cells were found to be reduced in the patient during the three treatment phases (Fig. 4). This may be due to less production of RBCs in bone marrow or their higher degradation in spleen. These results are consistent with previous studies [20, 21].

It has been suggested that the kidney may act as a sanctuary site for leukaemic cells in response to treatment with chlorambucil alone [22] which may explain our findings of worsening kidney function despite hematological remission was achieved after stopping the juice. Lymphocytic infiltration of the kidney may be considered as a possible cause. This result confirms that treatment with beetroot-carrot juice and chlorambucil was effective in the present case in protecting the kidneys. The betaine component of beetroot is as an organic osmolyte. It accumulates in the kidney [23] from exogenous origin to protect cells from high concentrations of electrolytes [24] and urea [25]. Beetroot-carrot juice may then be an effective treatment when given alone or in combination with chlorambucil, leading to improvement in renal function.

Our novel therapy leads the postulation that natural food product with known anticancer activity as beetroot-carrot juice, when used in right combination and dose could enhance the therapeutic efficacy of the potent antileukmic drug chlorambucil, by reducing its toxic side-effects. A possible synergistic effect of consumption of beetroot-carrot juice with chlorambucil resulted in a substantial improvement in renal function by acting as a kidney cleanser and protects the patient from renal impairement. Furthermore, beetroot-carrot juice could be used as natural alternative treatment for CLL when it was daily consumed as it has an antileukemic and anticancer effect.

Beetroot-carrot juice might have promising therapy strategies in treating B-CLL and that its combination with chlorambucil could improve the effectiveness of the drug and reduce its toxic side effects.

## References

[1] Goede V, Eichhorst B, Fischer K, Wendtner CM, Hallek M (2014). Past, present and future role of chlorambucil in the treatment of chronic lymphocytic leukemia. Leuk Lymphoma.

[2] Sonali K, Poonam K, Avinash M, Harish L (2014). Dietary source of anticancer agents. Int J Pharm Sci Rev Res.

[3] Nobili S, Lippi D, Witort E (2009). Natural compounds for cancer treatment and prevention. Pharmacological Research.

[4] David EL (1962). The red beetroot juice. Let’s live.

[5] Hartwell JL (1971). Plants used against cancer. A survey. Lloydia.

[6] Zaini R, Clench MR, Le Maitre CL (2011). Bioactive chemicals from carrot (Daucus carota) juice extracts for the treatment of leukemia. J Med Food.

[7] Hallek M (2014). A chemotherapy-free future? Novel treatment strategies for chronic lymphocytic leukemia Lancet. Hematology Education:the education program for the annual congress of the European Hematology Association.

[8] Sultana A, Islam A, Akhtar G, Rahman Md A (2012). Outcome of Tumor Lysis Syndrome with Hydration and Alkalinization in Children with Acute Lymphoblastic Leukemia. Bangladesh J Med Sci.

[9] Begleiter A, Lee K, Israels LG, Mowat MR, Johnston JB (1994). Chlorambucil induced apoptosis in chronic lymphocytic leukemia (CLL) and its relationship to clinical efficacy. Leukemia.

[10] Tomenendálová J, Mayer J, Doubek M (2008). Toxicity of High-Dose Chlorambucil in Wistar Rats. Acta Vet Brno.

[11] Junglee NA, Shrikanth S, Seale JR (2012). Rapidly progressive renal failure due to chronic lymphocytic leukemia -Response to chlorambucil. Indian J Nephrol.

[12] Lechner JF, Wang LS, Rocha CM (2010). Drinking water with red beetroot food color antagonizes esophageal carcinogenesis in N -nitrosomethylbenzylamine-treated rats. J Med Food.

[13] Boivin D, Lomy S, Lord-Dufour S (2009). Antiproliferative and antioxidant activities of common vegetables:A comparative study. Food Chem.

[14] Kapadia GJ, Rao GS, Ramachandran C (2013). Synergistic cytotoxicity of red beetroot (Betavulgaris L.) extract with doxorubicin in human pancreatic, breast and’ prostate cancer cell lines. J Complement Integr Med.

[15] Chavez-Santoscoy RA, Gutierrez-Uribe JA, Sema-Saldivar SO (2009). Phenolic composition, antioxidant capacity and in vitro cancer cell cytotoxicity of nine prickly pear (Opuntia spp) juices. Plant Food Hum Nutr.

[16] Sreekanth D, Arunasree MK, Roy KR, Chandramohan Reddy T, Reddy GV, Reddanna P (2007). Betanin, a betacyanin pigment purified from fruits of Opuntia fi cus-indica induces apoptosis in human chronic myeloid leukemia cell line-K562. Phytomedicine.

[17] Tripathy G, Pradhan D (2013). Evaluation of IN-VITRO anti-proliferative activity and IN-VIVO immunomodulatory activity of beta vulgaris. Asian J Pharm Clin Res.

[18] Kapadia GJ, Rao GS (2013). Anticancer effects of red beet pigments. Bhagyalaxmi Neelwarneed. Red Beet Biotechnology:Metabolites for food and pharmaceutical applications.

[19] Zaini R, Brandt K, Clench MR, Le Maitre CL (2012). Effects of bioactive compounds from Carrots (Daucus carota L.). Polyacetylenes, Beta-Carotene and Lutein on human lymphoid leukaemia cells. Anticancer Agents Med Chem.

[20] Al-abdallah O (2012). Effects of chronic myeloid leukemia on some haematological parameters and indicators during chemotherapy period. J Pharm Sci.

[21] Rafiq N, Iqbal T, Shahid M, Muhammad F (2014). Hematological and Biochemical Parameters in Pakistani Chronic Lymphoblastic Leukemia Patients. Pak J life soc sci.

[22] Phillips JK, Bass PS, Majumdar G, Davies DR, Jones NF, Pearson TC (1993). Renal failure caused by leukaemic infiltration in chronic lymphocytic leukaemia. J Clin Pathol.

[23] Pummer S, Dantzler WH, Lien YH, Moeckel GW, Volker K, Silbernagl S (2000). Reabsorption of betaine in Henle’s loops of rat kidney in vivo. Am J Physiol.

[24] Horio M, Ito A, Matsuoka Y (2001). Apoptosis induced by hypertonicity in Madin Darley canine kidney cells:protective effect of betaine. Nephrol Dial Transplant.

[25] Yancey PH, Burg MB (1990). Counteracting effects of urea and betaine in mammalian cells in culture. Am J Physiol.

